# Prognostic significance of the total number of harvested lymph nodes for lymph node-negative gastric cancer patients

**DOI:** 10.1186/s12885-017-3544-6

**Published:** 2017-08-22

**Authors:** Xin Ji, Zhao-De Bu, Zi-Yu Li, Ai-Wen Wu, Lian-Hai Zhang, Ji Zhang, Xiao-Jiang Wu, Xiang-Long Zong, Shuang-Xi Li, Fei Shan, Zi-Yu Jia, Jia-Fu Ji

**Affiliations:** Department of Gastrointestinal SurgeryKey Laboratory of Carcinogenesis and Translational Research (Ministry of Education), Peking University Cancer Hospital & Institute, No. 52 Fucheng Road, Haidian District, Beijing, 100142 China

**Keywords:** Number of harvested lymph nodes, Gastric cancer, Prognosis

## Abstract

**Background:**

The relationship between the number of harvested lymph nodes (HLNs) and prognosis of gastric cancer patients without an involvement of lymph nodes has not been well-evaluated. The objective of this study is to further explore this issue.

**Methods:**

We collected data from 399 gastric cancer patients between November 2006 and October 2011. All of them were without metastatic lymph nodes.

**Results:**

Survival analyses showed that statistically significant differences existed in the survival outcomes between the two groups allocated by the total number of HLNs ranging from 16 to 22. Therefore, we adopted 22 as the cut-off value of the total number of HLNs for grouping (group A: HLNs <22; group B: HLNs≥22). The intraoperative and postoperative characteristics, including operative blood loss (*P*=0.096), operation time (*P*=0.430), postoperative hospital stay (*P*=0.142), complications (*P*=0.552), rate of reoperation (*P*=0.966) and postoperative mortality (*P*=1.000), were comparable between the two groups. T-stage-stratified Kaplan–Meier analyses revealed that the 5-year survival rate of patients at the T4 stage was better in group B than in group A (76.9% vs. 58.5%; *P*=0.004). An analysis of multiple factors elucidated that the total number of HLNs, T stage, operation time and age were independently correlated factors of prognosis.

**Conclusions:**

Regarding gastric cancer patients without the involvement of lymph nodes, an HLN number ≥22 would be helpful in prolonging their overall survival, especially for those at T4 stage. The total number of HLNs was an independent prognostic factor for this population of patients.

## Background

Although recent advances in diagnostic techniques, surgical equipment and chemoradiotherapy have been rapidly developing, the incidence of gastric cancer still ranks fourth among all malignancies, and its mortality ranks second worldwide. According to recent reports, more than 950,000 patients with gastric cancer are identified each year, and more than 720,000 patients died from it in 2012 [1, 2].

Currently, surgical resection is the backbone of the cure for this disease. Gastrectomy combined with standard lymphadenectomy is a pivotal procedure. Despite standard lymphadenectomy being regarded as a crucial procedure of radical resection for curing gastric cancer, no consensus on the number of harvested lymph nodes (HLNs) has been achieved worldwide. The dissected lymph node number is influenced by many factors: degree of lymphadenectomy, surgical skills, conditions of the patients, examination technique used by pathologists, and so on. Presently, the numeric-based N staging system has been adopted by the East and West guidelines [3–5]. The National Comprehensive Cancer Network (NCCN) guidelines recommend that the total number of HLNs should be no less than 15 for an accurate nodal metastasis determination [3, 5]. The latest Japanese guidelines recommend that no less than 16 lymph nodes should be retrieved to accurately determine N staging [4]. Obviously, there is still controversy among different guidelines on how many lymph nodes should be dissected.

The request that a minimum number of HLNs be collected is to accurately determine N staging. Lymph node status is closely correlated with recurrence and prognosis. However, for patients without metastatic lymph nodes, a numeric-based N staging system contributes little to estimate their prognosis. Previous studies have reported that the number of HLNs potentially affects the prognosis of gastric cancer patients [6–8]. However, little is known about the minimum number of HLNs needed for a better survival outcome in N0 gastric cancer patients. In the light of the above-mentioned condition, the objective of our study is to determine the minimum number of HLNs needed for better survival outcomes for these patients and to determine the independent prognostic factors of these patients.

## Methods

### Patients

Approval from the ethics committee of our hospital and informed consent from each patient were acquired. We collected the clinicopathological and follow-up data from a database in our hospital. A total of 399 patients diagnosed as gastric adenocarcinoma with a pathological stage of N0 between November 2006 and October 2011 were included in our study. Each of them underwent radical resection and standard lymphadenectomy. The extent of the lymphadenectomy complied with the Japanese guidelines [9, 10]. The confirmation of diagnosis depended on the pathological examination. The clinical TNM staging was confirmed by upper gastrointestinal ultrasound endoscopy, abdominopelvic enhanced computed tomography scans, and laparoscopic exploration. The American Joint Commission for Cancer (AJCC) TNM classification (7th ed.) was applied in our study to classify the tumour stages. Patients who were diagnosed as other types of gastric carcinomas, such as lymphoma or gastric stromal tumours, were excluded from this study.

### Surgical procedure and recovery

All of the patients in this study underwent laparoscopic exploration and peritoneal lavage cytology examination to confirm that a distant metastatic disease did not exist. Subsequently, chief surgeons performed radical gastrectomies combined with standard lymph node dissections. The principle of surgical resection complied with the recommendations of the Japanese gastric cancer treatment guidelines [9, 10]. For patients with tumours limited to the T1 stage, a D1/D1+ lymphadenectomy was performed. For patients with tumour invasions to the T2 stage or deeper, a standard D2 lymphadenectomy was performed.

Perioperative chemotherapy was also recommended for some of the patients. According to the recommendations of the NCCN and Japanese guidelines, in our study, patients with a clinical stage of T3 or T4 were recommended to receive neoadjuvant chemotherapy (NACT), and those who were diagnosed with a pathological stage of T3 or T4 would receive adjuvant chemotherapy 4–6 weeks after the operation.

Medical care professionals would monitor the postoperative recovery of the patients. The patients had to stay in the hospital unless the discharge criteria were fulfilled, which included the following aspects: the absence of obvious subjective discomfort, recovery of gastrointestinal function and beginning of solid food intake, no need for parenteral nutrition or intravenous drugs, recovery of selfcare capacity and daily activities (e.g., capacity to eat, get dressed, take a bath alone, etc.), an adequate recovery of the wound, pulling out draining tubes, an adequate recovery from infectious or other postoperative complications, the return of normal clinical manifestations and laboratory examinations, an agreement to leave the hospital, and a satisfactory living environment.

### Clinicopathological data and follow-up

The data collected included the total number of HLNs, sex, age, height and weight, NACT, degree of differentiation, lymphovascular invasion (LVI), tumour size, depth of tumour invasion, extent of gastrectomy, operative bleeding, operation time, postoperative complications and mortality, reoperation, and length of postoperative hospital stay. During the first 5 years, the follow-up was carried out every 3 months, and after that it was carried out once every 6 months. Professional staff collected the follow-up information mainly by phone calls, emails, faxes or outpatient clinics. The follow-up continued until November 2016.

### Statistical analysis

IBM SPSS Statistics 20.0 software (SPSS Inc., Armonk, NY, USA) was used to analyse the data. Chi-squared tests or Fisher’s exact tests were applied to analyse the categorical data. With regards to quantitative variables, they were analysed by *t* tests and expressed as the mean ± standard deviation (SD) if a normal distribution was verified. Otherwise, they would be expressed as the median with an interquartile range (IQR, 25th and 75th percentiles), and Kruskal–Wallis non-parametric tests would be performed. Survival outcomes were compared using Kaplan–Meier survival analysis. The factors that were correlated with prognosis were verified using Cox regression analysis. A *P* < 0.05 (two-sided) indicated a significant difference.

## Results

### Cut-off point analysis of the total number of HLNs

**Fig. 1:**
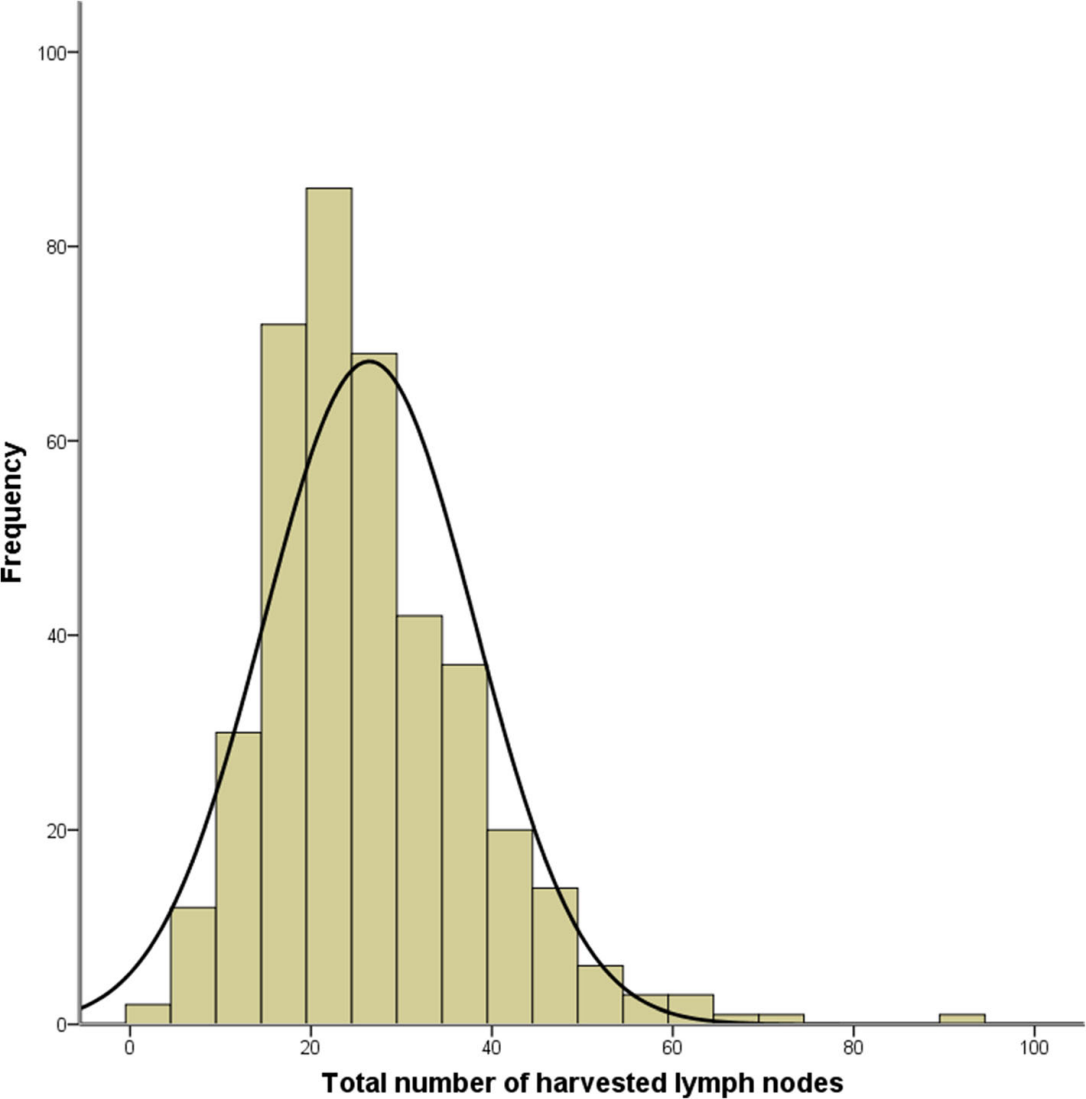
The frequency distribution of the harvested lymph nodes for all the patients. The median number of HLNs for all the patients was 24 (IQR: 19–33)

The median number of HLNs of all of the patients was 24 (IQR: 19–33). The results of the distribution are listed in Fig. 1. We allocated patients into two groups according to the cut-off numbers of HLNs from 16 to 50. In every cut-off value, we compared the survival outcomes between the two groups. The analyses elucidated that the survival outcomes between the two groups were significantly different when the cut-off value of HLNs ranged from 16 to 22 (Table 1). Thus, we allocated the patients into two groups based on this result. In groups A and B, the total numbers of HLNs were <22 and ≥22, respectively.

**Table 1:** The prognostic impact in N0 gastric cancer patients depending on different cut-off numbers of HLNs

Cut-off number of HLNs	*P-*value^a^
16	**<0.001**
17	**0.002**
18	**0.039**
19	**0.008**
20	**0.002**
21	**0.013**
22	**0.047**
23	0.144
24	0.164
25	0.098
26	0.238
27	0.305
28	0.807
29	0.858
30	0.596
31	0.428
32	0.209
33	0.266
34	0.201
35	0.446
36	0.499
37	0.537
38	0.223
39	0.803
40	0.728
41	0.619
42	0.842
43	0.998
44	0.934
45	0.826
46	0.628
47	0.387
48	0.508
49	0.501
50	0.783

HLNs: harvested lymph nodes

^a^ *P-*values were calculated by the log-rank test for survival curves that were generated by the Kaplan–Meier method, and the statistically significant values (*P* < 0.05) are in bold

### Clinicopathological parameters

Altogether, we analysed the data of 399 patients. They were allocated into either group A (HLNs <22; *n* = 157) or group B (HLNs ≥22; *n* = 242). The clinicopathological factors were compared between the two groups. The analyses revealed that body mass index (BMI), age, sex, the rate of receiving NACT, and LVI were comparable between the groups. The tumour size was smaller in group A (*P* < 0.001). Compared with the patients in group A, more patients in group B were diagnosed with poorly differentiated tumours (*P* = 0.003) and more patients in group B underwent total gastrectomy (*P* < 0.001). Moreover, more patients in group B were diagnosed with a relatively later T stage than those in group A (*P* = 0.003; Table 2).

**Table 2:** The patients’ clinicopathological parameters and comparisons of them between the two groups

Clinicopathological parameters	Group A (HLNs <22; *N* = 157), *n* (%)	Group B (HLNs ≥22; *N* = 242), *n* (%)	*P-*value
Sex			0.504
Male	110 (70.1)	177 (73.1)	
Female	47 (29.9)	65 (26.9)	
Age			0.108
≤60	89 (56.7)	147 (60.7)	
>60	68 (43.3)	95 (39.3)	
BMI			0.732
<19	13 (8.4)	18 (7.7)	
~ < 25	93 (60.4)	154 (65.5)	
~ < 30	43 (27.9)	58 (24.7)	
≥30	5 (3.2)	5 (2.1)	
NACT			0.392
No	114 (72.6)	166 (68.6)	
Yes	43 (27.4)	76 (31.4)	
Differentiation			0.003
Well	12 (8.5)	11 (4.8)	
Moderate	90 (63.4)	113 (49.8)	
Poor	40 (28.2)	103 (45.4)	
LVI			0.834
No	139 (88.5)	213 (88.0)	
Yes	18 (11.5)	29 (12.0)	
Tumour size			<0.001
≤5 cm	146 (95.4)	196 (83.4)	
>5 cm	7 (4.6)	39 (16.6)	
Gastrectomy			<0.001
Distal	84 (54.2)	145 (59.9)	
Proximal	48 (31.0)	23 (9.5)	
Total	23 (14.8)	74 (30.6)	
T stage^a^			0.003
T1	65 (43.6)	64 (27.6)	
T2	37 (24.8)	53 (22.8)	
T3	1 (0.7)	2 (0.9)	
T4	46 (30.9)	113 (48.7)	

*HLNs* harvested lymph nodes, *BMI* body mass index, *NACT* neoadjuvant chemotherapy, *LVI* lymphovascular invasion

^a^7^th^ AJCC TNM staging system for gastric cancer

### Intraoperative and postoperative parameters

We also compared surgery-related parameters between the two groups. The results showed that operation time, operative bleeding, complication, mortality, rate of reoperation, and postoperative hospital stay were similar in both groups (Table 3).

**Table 3:** The patients’ intraoperative and postoperative parameters and comparisons of them between the two groups

Intraoperative and postoperative parameters	Group A (HLNs <22; *N* = 157)	Group B (HLNs ≥22; *N* = 242)	*P-*value
Operation duration, min, median (IQR)	200 (175–250)	200 (165–240)	0.430
Blood loss volume, mL, median (IQR)	100 (100–200)	100 (100–200)	0.096
Postoperative hospital stay, days, median (IQR)	12 (10–17)	12 (10–15)	0.142
Complication rate, *n* (%)	20 (12.7)	26 (10.8)	0.552
Reoperation, *n* (%)	4 (2.5)	6 (2.5)	0.966
Mortality rate, *n* (%)	0 (0.0)	0 (0.0)	1.000

*HLNs* harvested lymph nodes, *IQR* interquartile range (25th and 75th percentiles)

### Survival outcomes

**Fig. 2:**
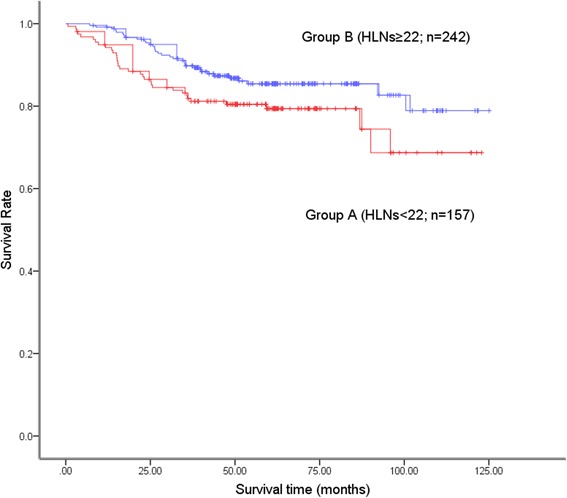
The overall survival curves of the patients in group A (HLNs <22) and group B (HLNs ≥22). The overall survival was better in group B than in group A (*P* = 0.047). The 5-year survival rates in group A and group B were 79.4% and 85.4%, respectively

**Fig. 3:**
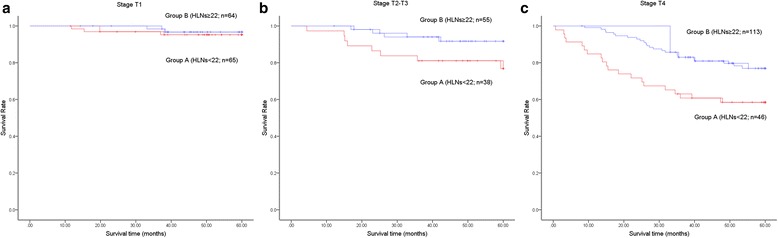
The stage-stratified survival curves of patients in the two groups. (**a**) In the stage-stratified subgroup analysis of patients at stage T1, the 5-year survival rates in group A and group B were 95.2% and 96.7%, respectively (*P* = 0.641). (**b**) For the patients at stages T2–T3, the 5-year survival rates in group A and group B were 76.8% and 91.7%, respectively (*P* = 0.066). (**c**) In the subgroup analysis of patients at stage T4, the 5-year survival rates in group A and group B were 58.5% and 76.9%, respectively (*P* = 0.004)

Until 11 November 2016, the follow-up varied from 1 to 125 months (median: 58.0 months). The analysis showed that the patients in group B had a better 5-year survival rate than those in group A (85.4% vs. 79.4%; *P* = 0.047; Fig. 2). Considering the clinicopathological differences that existed between the two groups, we conducted subgroup analyses according to the T stage. In the stratified analyses of patients at stage T1 and stages T2–T3, the 5-year survival rates of patients were comparable in both groups (Fig. 3a; Fig. 3b). With regarding to patients at stage T4, patients in group B had a better 5-year survival rate than those in group A (76.9% vs. 58.5%; *P* = 0.004; Fig. 3c).

All of the clinicopathological and surgery-related parameters were included in the univariate analysis. Tumour size, the extent of the gastrectomy, operative bleeding, postoperative complications, age, NACT, operation duration, T stage and total number of HLNs were associated with the prognosis (Table 4). Next, the aforementioned parameters were included into a multiple factors analysis. The results showed that the independent prognostic factors included age, operation duration, T stage and total number of HLNs (Table 4).

**Table 4:** The univariate and multivariate analyses of the prognostic factors for survival

	Univariate HR (95% CI)	*P*-value	Multivariate HR (95% CI)	*P*-value
Sex		0.138		
Male	1			
Female	1.462 (0.883, 2.421)			
BMI		0.437		
<19	1			
~ < 25	0.901 (0.383, 2.116)			
~ < 30	0.671 (0.258, 1.745)			
≥30	0.891 (0.128, 1985)			
Differentiation		0.284		
Well	1			
Moderate	2.063 (0.495, 8.587)			
Poor	2.669 (0.637, 11.188)			
LVI		0.659		
No	1			
Yes	1.336 (0.681, 2.622)			
Postoperative hospital stay	1.004 (0.996, 1.013)	0.335		
Reoperation		0.247		
No	1			
Yes	1.959 (0.614, 6.246)			
Tumour size		0.013		0.643
≤5 cm	1		1	
>5 cm	2.088 (1.151, 3.789)		1.174 (0.596, 2.314)	
Gastrectomy		<0.001		0.707
Distal	1		1	
Proximal	2.565 (1.392, 4.728)		1.267 (0.649, 2.473)	
Total	2.746 (1.555, 4.848)		1.267 (0.665, 2.413)	
Blood loss volume	1.001 (1.001, 1.002)	0.001	1.001 (0.999, 1.002)	0.296
Complication		0.013		0.332
No	1		1	
Yes	2.122 (1.157, 3.890)		1.387 (0.716, 2.686)	
Age		0.009		0.031
≤60	1		1	
>60	1.918 (1.169, 3.148)		1.830 (1.058, 3.165)	
NACT		0.002		0.093
No	1		1	
Yes	2.133 (1.310, 3.473)		1.600 (0.924, 2.771)	
T stage^a^		<0.001		<0.001
T1	1		1	
T2	4.126 (1.486, 11.461)		3.930 (1.378, 11.209)	
T3	6.065 (2.463, 16.435)		5.089 (2.456, 15.364)	
T4	8.092 (3.211, 20.391)		7.946 (3.065, 20.691)	
Operation duration	1.006 (1.003, 1.008)	<0.001	1.007 (1.004, 1.010)	<0.001
Total number of HLNs		0.047		0.002
*N* < 22	1		1	
*N* ≥ 22	0.618 (0.383, 0.999)		0.437 (0.262, 0.730)	

*HLNs* harvested lymph nodes, *HR* hazard ratio, *CI* confidence interval, *BMI* body mass index, *LVI* lymphovascular invasion, *NACT* neoadjuvant chemotherapy

^a^7^th^ AJCC TNM staging system for gastric cancer

## Discussion

The argument concerning the minimum number of HLNs has continued for a long time among the various world regions [11]. Eastern surgeons believed that extended lymphadenectomy with more dissected lymph nodes would bring therapeutic benefit for gastric cancer patients [12]. In contrast, Western surgeons regarded extended lymphadenectomy as an unnecessary procedure [13, 14]. Recently, however, the long-term results of a trial from Netherlands demonstrated that D2 lymphadenectomy decreased the rate of locoregional recurrence and improved the overall survival of patients [15]. Based on this evidence, the importance of extended lymphadenectomy and a sufficient number of HLNs was again confirmed. However, the exact minimum number of HLNs has not been identified to date.

In our study, patients with an HLN number of 22 or more have a better survival. The phenomenon that an increased number of HLNs could be helpful in prolonging survival in gastric cancer patients without involvement of the lymph nodes has been reported before, while the minimum number of HLNs has not been confirmed [16, 17]. The explanations for this phenomenon are as follows. First, an insufficient number of HLNs might miss potentially metastatic lymph nodes, which could induce a recurrence of the tumour. Second, an insufficient number of HLNs could induce stage deviation and guide incorrect adjuvant therapeutic decisions [18]. Third, previous studies reported that LVI might induce poor survival outcomes in gastric cancer patients [19, 20]. An adequate number of HLNs might remove the lymph nodes and lymphatic vessels that have potential LVI and reduce local recurrence of the cancer.

The clinicopathological parameters of the patients were compared between the two groups. In group B (HLNs ≥22), more patients underwent total gastrectomy and stayed in a relatively later T stage. A possible reason for this is that standard D2 lymphadenectomy was required for locally advanced gastric cancer, which should harvest more lymph nodes than D1/D1+ lymphadenectomy. Standard D2 lymphadenectomy in total gastrectomy includes more groups of lymph nodes than that in distal or proximal subtotal gastrectomy.

In addition, a greater number of HLNs had no negative effect on the perioperative safety and recovery. These results demonstrated that a greater number of HLNs did not increase the risks of the operation. The primary reason for this was that all of the chief surgeons in our centre were experienced surgeons and the operation procedures of our centre strictly complied with the Japanese guidelines. Those complications were possibly related to the surgeons’ skills and the perioperative management rather than to the extent of lymphadenectomy. Therefore, the standard lymphadenectomy did not increase the risk of postoperative complications, just as previous studies have reported [13, 15].

Considering the imbalance of the T stages between the two groups, we compared the survival outcome through stratified analyses according to the T stage. Significant differences were not found in the T1 and T2–T3 stages subgroup analyses. However, for T4 stage disease, the 5-year survival rate was better in group B than in group A (76.9% vs. 58.5%; *P* = 0.004). The increased number of HLNs (≥22) showed a survival benefit in T4-stage patients. This result might be correlated with the rate of lymph node involvement at different depths of tumour invasion. Previous studies have reported that the percentage of lymph node metastasis at T1 stage was no more than 10%. When the tumour invaded a deeper layer of the stomach, the risk of lymph node involvement greatly increased [21]. An insufficient number of HLNs failed to affect the survival outcome at the T1-stage and T2- to T3-stage patients for the reason that initially involved lymph nodes, which might induce recurrence, were rare. However, an increased number of HLNs was found to be helpful in prolonging survival in gastric cancer patients at the T4 stage.

The results of the multiple factor analysis demonstrated that the independent prognostic factors included age, operation time, T stage and the total number of HLNs (Table 4). In accordance with the TNM staging system, tumour invasion was an independent correlated factor of prognosis. Moreover, the total number of HLNs was also an independent correlated factor of prognosis. Some previous studies had arrived at similar conclusions [17, 22, 23]. Because the total number of HLNs can be controlled by surgeons and pathological diagnostic techniques, it is essential that surgeons increase the total number of HLNs during operations.

Several limitations of the present study exist. First, a selection bias exists because it is a retrospective observational study. Second, because of the limitations of the follow-up, the information regarding detailed postoperative chemotherapy regimens and cycles was not collected, which might affect the results of the survival analysis. However, the T-stage-stratified subgroup and multiple factors analyses were performed to neutralize the confounding factors and the selection bias. The results of this study thus remain persuasive. To further investigate this issue, a randomized controlled clinical trial is necessary to evaluate the relationship between prognosis and the total number of HLNs.

## Conclusions

For gastric cancer patients who are free of the involvement of lymph nodes, a total number of HLNs ≥22 would be helpful for prolonging their overall survival. Especially for patients at the T4 stage, a total number of HLNs ≥22 is strongly recommended. The total number of HLNs was an independent prognostic factor of gastric cancer in these patients.

## Abbreviations

AJCC: American Joint Commission for Cancer
BMI: Body mass index
HLNs: Harvested lymph nodes
IQR: Interquartile range
LVI: Lymphovascular invasion
NACT: Neoadjuvant chemotherapy
NCCN: National Comprehensive Cancer Network
SD: Standard deviation
